# Contractile effect of Sclerocarya *birrea* (A Rich) Hochst (Anacardiaceae) (Marula) leaf aqueous extract on rat and rabbit isolated vascular smooth muscles

**DOI:** 10.5830/CVJA-2010-098

**Published:** 2012-02

**Authors:** Tariro Mawoza, John AO Ojewole, Peter MO Owira

**Affiliations:** Department of Pharmacology, School of Pharmacy and Pharmacology, Faculty of Health Sciences, University of KwaZulu-Natal, Durban, South Africa; Department of Pharmacology, School of Pharmacy and Pharmacology, Faculty of Health Sciences, University of KwaZulu-Natal, Durban, South Africa; Department of Pharmacology, School of Pharmacy and Pharmacology, Faculty of Health Sciences, University of KwaZulu-Natal, Durban, South Africa

**Keywords:** *Sclerocarya birrea*, contraction, vascular smooth muscles

## Abstract

**Backround:**

*Sclerocarya birrea* (Anacardiaceae) is traditionally used for treating hypertension. The pharmacological effects of *S birrea* leaf aqueous extract (SBE) on rabbit and rat vascular smooth muscles were investigated in this study.

**Methods:**

Fresh S *birrea* leaves (1 kg) were air dried at 26 ± 1°C, milled, macerated in 2.5 l of distilled water for 48 hours, filtered, and the filtrate was concentrated in a rotary evaporator. Rat isolated portal vein preparations, as well as rabbit isolated endothelium-denuded and endothelium-intact descending thoracic aortic ring preparations were mounted in 30-ml Ugo Basile organ baths under physiological conditions, and challenged with SBE (50–400 mg/ml). The contractile effects of SBE and/or other reference drugs on the isolated vascular smooth muscle preparations were recorded by means of Ugo Basile’s force–displacement transducers and Gemini recorders.

**Results:**

SBE (50–400 mg/ml) caused a significant, concentration-dependent upward shift in baseline tone in the aortic ring preparations (*p* < 0.01–0.001). Indomethacin (20 µM) markedly attenuated the contractile effects of SBE in both the endothelium-intact and -denuded aortic rings, while N^G^-nitro-_L_-arginine methyl ester (L-NAME, 100 µM) significantly (*p* < 0.05) increased the contractile tension of the endothelium-intact aortic rings. Verapamil (1–3 µg/ml) partially inhibited the contractile effects of SBE. SBE also elicited significant (*p* < 0.05–0.01) increases in the amplitude of the myogenic contractions of the portal veins. These contractions were abolished by verapamil (1–3 µg/ml) in a concentration-dependent manner, while prazosin (1–3 µg/ml) did not affect the SBE-induced contractions.

**Conclusion:**

SBE possessed spasmogenic effects on vascular smooth muscle preparations *in vitro*. It may induce and/or exacerbate hypertension, contrary to the folkloric, ethnomedical use of S *birrea*.

## Abstract

*Sclerocarya birrea* (A Rich) Hochst (family: Anacardiaceae), popularly known as the cider or marula tree in English, *maroele* in Afrikaans, or *umganu* in IsiZulu, is a medium-sized, single-stemmed, perennial, deciduous tree with grey-fissured bark, stout branchlets and pale foliage.^1^ The tree is native to sub-Saharan Africa,^2^ with different species distributed from Ethiopia to KwaZulu-Natal Province in South Africa,^3^ where it is widely used by local communities as a source of food and for ethnomedical as well as cultural practices.[Bibr R04]-6

In South Africa, the stem bark, roots and leaves of *S birrea* have traditionally been used to treat human ailments such as infections (malaria, fever, diarrhoea, dysentery, schistosomiasis) and degenerative diseases (stomach disorders, headaches, toothache, high blood pressure, backache, dysmenorrhoea, body pains, diabetes mellitus, arthritis).^7^-^10^ The Zulus of South Africa have also used decoctions of *S birrea* stem bark and leaf aqueous extracts as enemas for diarrhoea and for prophylaxis against gangrenous rectitis,^8^,^11^ dysentery, fevers, stomach ailments, ulcers and bacterial-related diseases.^11^ The Vhavenda of Limpopo Province in South Africa reportedly use the stem bark of *S birrea* for treating fevers, stomach ailments and ulcers.^12^ In other rural African communities, chewing fresh leaves of *S birrea* and swallowing the astringent juice has been reported to help with indigestion.^9^

Ethnomedicinal use of the extracts of *S birrea* in the treatment of stomach ailments and high blood pressure, among others, suggests that certain chemical constituents in these extracts may affect smooth muscle contractility. In an attempt to provide a pharmacological rationale (or otherwise) for the folkloric and ethnomedicinal uses of the extracts of this plant in the treatment of diseases affecting vascular smooth muscles, the present study was undertaken to investigate the contractile effects of *S birrea* leaf aqueous extract (SBE) on mammalian isolated vascular smooth muscles. We hypothesised that if SBE possesses antihypertensive effects, this would justify its folkloric use in the management of hypertension. If, however, SBE induces contractile effects on vascular smooth muscles, it would be contraindicated in hypertensive patients.

## Methods

Experimental protocols and procedures used in this study were approved by the Animal Ethics Committee of the University of KwaZulu-Natal and conformed to the *Guide to the Care and Use of Animals in Research and Teaching*, published by the Animal Ethics Committee of the University of KwaZulu-Natal, Durban, South Africa.

Fresh leaves of *S birrea* were collected from an open grassland field on the Westville campus of the University of KwaZulu-Natal, Durban. Identification and authentication of the plant material were done by the taxonomist/curator of the Botany Department, University of KwaZulu-Natal, voucher specimen no. 685.

Fresh leaves (1 kg) of *S birrea* were air dried at room temperature (26 ± 1°C) for two weeks. They were then milled in a Waring commercial blender into fine powder, and macerated in 2.5 l of distilled water, with occasional shaking, for 48 hours at room temperature. The powdered leaves were extracted twice with 2.5 l of distilled water on each occasion, before being filtered. A rotary evaporator was used to concentrate the aqueous extract (filtrate) *in vacuo* at 60 ± 1°C. Freeze-drying and solvent elimination of the resulting aqueous filtrate produced 55.5 g (i.e. 5.55% yield) of a light-brown, powdery, crude leaf extract. Without any further purification, portions of this extract were weighed and dissolved in distilled water (at room temperature) for use on each day of our experiments.

Healthy young adult, treatment-naïve, male and female Wistar rats (180–200 g, *n* = 40) and New Zealand white albino rabbits (1.5–3.0 kg, *n* = 6) were used. The animals were kept under conventional laboratory conditions of temperature, humidity and light, and allowed free access to food (standard pellet diet) and tap water. All the animals were fasted for 16 hours, but allowed free access to drinking water before the commencement of our experiments.

Each rat was euthanised by halothane inhalation. The rabbits were anaesthetised by intramuscular administration of a ketamine (30 mg/kg body weight) and xylazine (5 mg/kg body weight) mixture, followed by intravenous injection of 200 mg/kg body weight of sodium pentobarbital (Euthapent®) through the ear vein. The abdomen and thorax were then opened via midline incision and the descending thoracic aorta of the rabbits and the portal vein of the rats were quickly removed.

## Rat isolated aortic rings

Isolated descending thoracic aortic ring preparations (3 mm long) and isolated portal veins were suspended in 30-ml Ugo Basile organ baths containing Krebs-Henseleit physiological solution (KHS) of composition (in mM): NaCl, 118; KCl, 4.7; NaHCO_3_, 25; MgSO_4_, 1.2; CaCl_2_.2H_2_O, 2.52; KH_2_PO_4_.2H_2_O, 1.28; and D-glucose, 5.55 (pH adjusted to 7.4). Calcium-free KHS was made by adding 0.2 mM ethylenediaminetetra-acetic acid (EDTA) to the physiological solution. The bathing KHS was maintained at 35 ± 1°C and aerated continuously with carbogen, (95% O_2_ + 5% CO_2_).

Two smooth muscle preparations from the same animal (one used as control and the other as SBE and/or reference drug-treated test preparation) were always set up at a time in order to make allowances for changes in smooth muscle sensitivity. The tissues were maintained in 30-ml Ugo Basile organ baths (under physiological conditions) for 45–60 minutes to equilibrate, during which time the bathing KHS was changed every 15 minutes.

After the equilibration period, graded concentrations of SBE (50–400 mg/ml) and/or the reference drugs were added to the bath fluid sequentially at 10-minute intervals. Where necessary, bath-applied SBE and/or reference drug concentrations were repeated after washing out the previous SBE (and/or reference drug concentration/s) four to five times and then allowing the tissue to rest for five to 10 minutes, or until its tone returned to baseline. The control smooth muscle tissue preparations were treated with distilled water (0.5–4 ml only, equivalent to the volumes of bath-applied SBE or reference drug solution used). Each test smooth muscle preparation was used for one concentration–response curve only.

## Rabbit isolated aortic ring

Each isolated descending thoracic aortic ring preparation was subjected to the same experimental conditions as for the rat aortic rings and portal veins described above. In some of the rabbit aortic ring preparations, the endothelium was removed mechanically *in situ* by rubbing the luminal surfaces gently six to eight times with a glass rod.

In order to characterise the involvement of the nitric oxide pathway prior to the addition of SBE (50–400 mg/ml), some of the endothelium-intact aortic rings were pre-incubated with N^G^-nitro-_L_-arginine-methyl-ester (L-NAME, 100 µM), a nitric oxide synthase inhibitor. In addition to verapamil (1–3 µg/ml), an L-type calcium channel blocker, Ca^2+^-free KHS was used to test the contractile effects of SBE.

The possible role of indomethacin (20 µM), a cyclo-oxygenase pathway inhibitor, on the SBE-induced contractile response was also examined in rabbit aortic ring preparations. The effect of SBE and/or other reference drugs on the aortic ring preparations was recorded by means of Ugo Basile’s force–displacement transducers and pen-writing, two-channel Gemini recorders.

## Rat isolated portal vein preparations

Each rat isolated portal vein preparation was suspended in a 30-ml Ugo Basile organ bath containing KHS, and subjected to the same experimental conditions as for the aortic rings above. In addition to verapamil (1–3 µg/ml), Ca^2+^-free KHS was used to test the contractile effects of SBE (50–400 mg/ml). The possible role of prazosin (1–3 µg/ml), an α_1_-adrenergic receptor blocker, on the SBE-induced contractile responses was also investigated in some isolated portal vein preparations. Six of the animals used in this study were pre-treated with reserpine (1 mg/kg subcutaneously), a catecholamine blocker, to deplete catecholamines from the neuronal stores and subsequently block their uptake.

## Statistical analysis

Contractile responses to graded concentrations of SBE and/ or reference drugs on the different tissues were calculated as per-gram increments of maximal contractions (1 g = 2 cm). Experimental data were obtained as three to four duplicates, using data from different animals where applicable, and presented as means [± standard error of the means (SEM)] of measurements.

Data from distilled water-treated control smooth muscle preparations were used as baseline values. In all cases, data from the SBE- and/or reference drug-treated test smooth muscle preparations were compared with those from distilled watertreated controls.

Using GraphPad Prism® Version 5.00 (GraphPad® Software, Inc. San Diego, Califonia), the differences between the data obtained from test and control smooth muscle preparations were analysed for statistical significance, using Student’s *t*-test, and/or one-way analysis of variance (ANOVA; 95% confidence interval) for multiple value comparison, followed by Dunnett’s *post hoc* test. In all cases, values of *p* ≤ 0.05 were taken to imply statistical significance. To obtain the concentrations of SBE producing 50% of the maximal stimulant effect (EC_50_), concentration–response slopes were analysed using linear regression analysis.

## Results

### Effect of SBE on rabbit isolated aortic ring strips

**Fig. 1:**
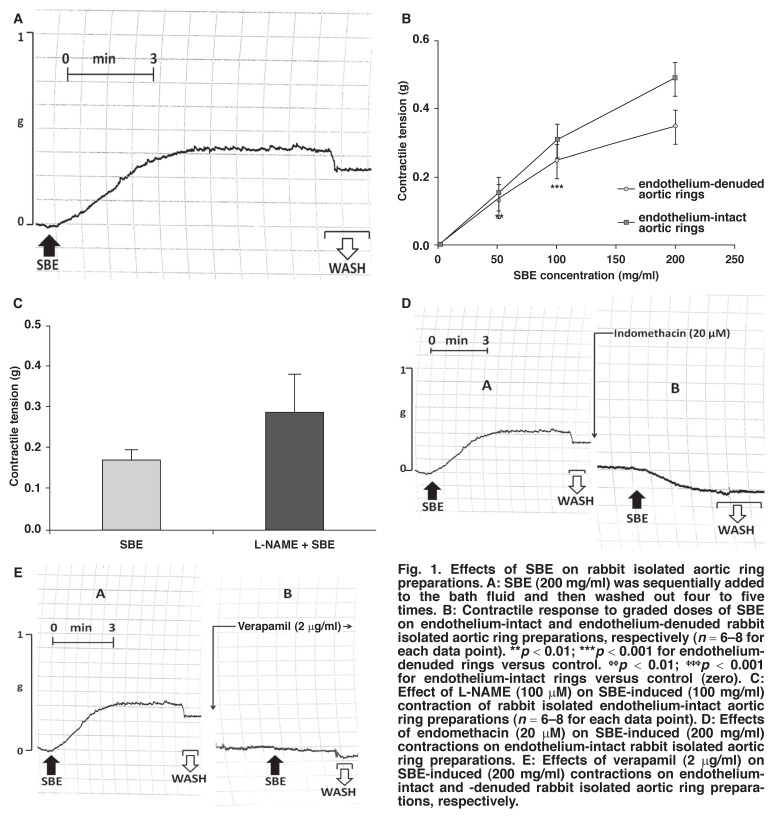
Effects of SBE on rabbit isolated aortic ring preparations. A: SBE (200 mg/ml) was sequentially added to the bath fluid and then washed out four to five times. B: Contractile response to graded doses of SBE on endothelium-intact and endothelium-denuded rabbit isolated aortic ring preparations, respectively (*n* = 6–8 for each data point). **p < 0.01; ***p < 0.001 for endotheliumdenuded rings versus control. ^ϕϕ^*p* < 0.01; ^ϕϕϕ^*p* < 0.001 for endothelium-intact rings versus control (zero). C: Effect of L-NAME (100 µM) on SBE-induced (100 mg/ml) contraction of rabbit isolated endothelium-intact aortic ring preparations (*n* = 6–8 for each data point). D: Effects of endomethacin (20 µM) on SBE-induced (200 mg/ml) contractions on endothelium-intact rabbit isolated aortic ring preparations. E: Effects of verapamil (2 µg/ml) on SBE-induced (200 mg/ml) contractions on endotheliumintact and -denuded rabbit isolated aortic ring preparations, respectively.

Incubation of aortic rings, harvested from normal rabbits, with SBE (50–400 mg/ml) resulted in a significant (*p* < 0.01–0.001) upward shift in baseline tone, with a mean positive baseline shift of 0.56 ± 0.05 g (*n* = 6–8) in endothelium-intact, and 0.45 ± 0.03 g (*n* = 6–8) in endothelium-denuded aortic ring preparations (Fig. 1A). Removal of the functional endothelium significantly (*p* < 0.01) increased the SBE-induced contractile responses of the preparations (Fig. 1B). The EC_50_ of SBE’s contractile effects on the endothelium-intact and endothelium-denuded aortic rings were calculated to be 92 ± 3 mg/ml and 93 ± 5 mg/ml, respectively.

The contractile effects of SBE were partially reversed by washing out the SBE solutions and then allowing the tissue to rest for five to 10 minutes (Fig. 1A). The contractile tension of the endothelium-intact aortic rings in response to SBE was increased by L-NAME (100 µM) (Fig. 1C), but not significantly (*p* > 0.05), compared with SBE-only treatment.

Pre-incubation of the aortic rings with indomethacin (20 µM) markedly inhibited or abolished the contractile effects of SBE and this was characterised by a downward shift in baseline tone (Fig. 1D). No visible change in baseline tone was observed with SBE in Ca^2+^-free KHS. Pre-incubation of the tissues with verapamil (1–3 µg/ml), however, partially inhibited the contractile effects of SBE in both endothelium-intact and -denuded rabbit aortic ring preparations (Fig. 1E).

### Effect of SBE on rat isolated portal veins

**Fig. 2:**
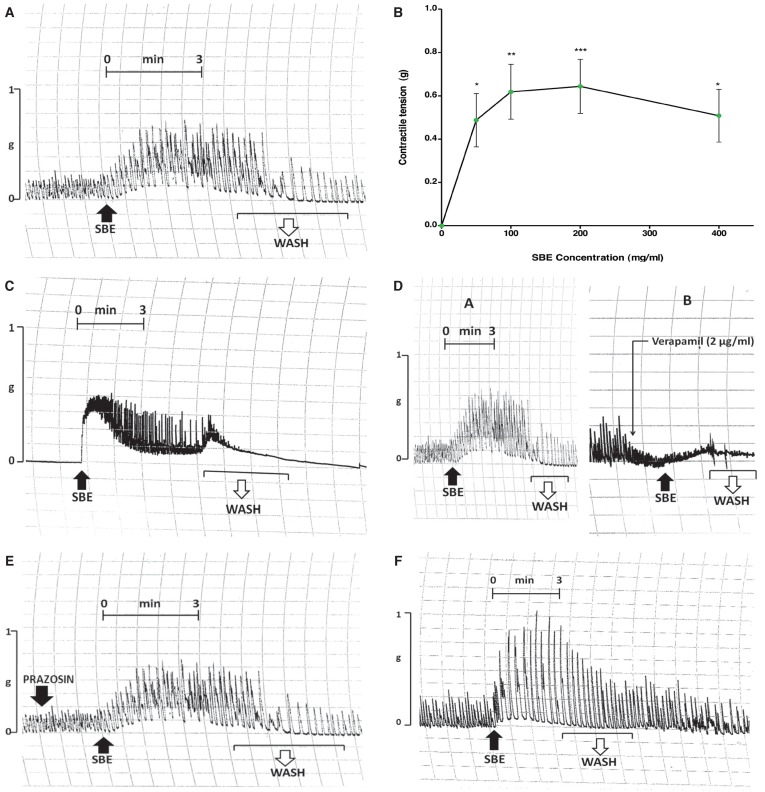
Effects of SBE on rat isolated portal vein preparations. A: SBE (100 mg/ml) was sequentially added to the bath fluid and then washed out four to five times. B: Contractile response to graded concentrations of SBE. *p < 0.05; **p < 0.01; ***p < 0.001 versus control (zero). C: Contractile responses to SBE (200 mg/ml) in Ca^2+^-free Krebs-Henseleit physiological solution. D: Effect of verapamil (2 µg/ml) on SBE-induced (100 mg/ml) contractions. E: Pre-incubation of isolated tissue with prazosin (1–3 µg/ml) followed by SBE administration. F: Contractile responses to SBE (100 mg/ml) in reserpine pre-treated tissues.

The rat portal veins exhibited spontaneous rhythmic, myogenic contractions with a mean amplitude of 0.89 ± 0.11 g (*n* = 6–8). SBE raised the baseline tone and subsequently caused marked (*p* < 0.05–0.01) concentration-dependent increases in the amplitude of the contractions (Fig. 2A, B). The EC_50_ concentration of SBE’s contractile effect on the preparations was calculated to be 36 ± 6 mg/ml (*n* = 6–8). The contractile effects of SBE were partially reversed by washing out the SBE solutions and allowing the tissue to rest for five to 10 minutes.

In Ca^2+^-free KHS, the portal vein preparations were devoid of contractions. SBE sequentially added to the bath fluid caused significant concentration-related increases (*p* < 0.05–0.001) in the baseline tone, and induced powerful rhythmic contractions (Fig. 2C). These contractile effects were completely reversed by washing out the SBE (four to five times) and allowing the venous tissue to rest for five to 10 minutes.

Pre-incubating the tissue with verapamil (1–3 µg/ml) profoundly attenuated the contractile effects of SBE in a concentration-dependent manner, and subsequently decreased the contractile amplitudes of the portal vein preparations, although an upward shift in baseline tone was still observed (Fig. 2D). Pre-incubation of the isolated venous tissue with prazosin (1–3 µg/ml), on the other hand, did not affect the SBE-induced contractions (Fig. 2E).

### Effect of SBE on reserpinised rat isolated portal veins

Rat isolated portal vein preparations which were taken from reserpine pre-treated animals (1 mg/kg subcutaneously) exhibited spontaneous, rhythmic, myogenic contractions. SBE sequentially added to the bath fluid caused significant concentrationdependent increases (*p* < 0.05–0.001) in the baseline tone. The contractile effects of SBE were partially reversed by washing it out and allowing the tissue to rest for five to 10 minutes. However, the contractile effects of SBE on the rat portal veins were not modified by prior reserpinisation of the animals (Fig. 2F).

## Discussion

The findings of this study show that SBE possessed contractile effects on rabbit isolated aortic ring preparations. Graded concentrations of SBE (50–200 mg/ml) markedly (*p* < 0.01–0.001) contracted freshly mounted, treatment-naïve, endothelium-intact aortic ring preparations (Fig. 1B) in a concentration-dependent manner, with an EC_50_ value of 92 ± 3 mg/ml.

These results contradict earlier observations of Ojewole,^13^ in which an aqueous stem bark extract of *S birrea* produced significant concentration-dependent relaxation (*p* < 0.05–0.001) of rat isolated endothelium-intact aortic ring preparations. This was attributed to the formation and release of endothelium-derived nitric oxide by the phytochemicals present in SBE. However, the present study reports for the first time that SBE induced contractions in vascular smooth muscles.

The discrepancy in the findings of these two studies could be due to the differences in the animal species and the morphological parts of the plant used. The differences could also be due to variability in the extracts, considering that not all of the bioactive components are extractable in water.

In our study, addition of L-NAME to the bath fluid 10 minutes prior to treatment of the rabbit endothelium-intact aortic ring preparations with SBE increased the SBE-induced contractile effects (Fig. 1C), compared to SBE-only treatment, but the differences were not statistically significant. The increase in contractility could be attributed to the inhibitory effect of L-NAME on nitric oxide (NO) synthesis. The presence of endothelium-derived NO could have partially reduced the SBE-induced contractile effects in the endothelium-intact aortic ring preparations. NO is a potent vasodilator and vasorelaxant,^14^,^15^ and inhibition of its synthesis would result in an increase in the contractile effects of certain compounds; hence the increase in SBE-induced vasoconstriction in tissues pre-incubated with L-NAME.

On the other hand, pre-incubating aortic ring preparations with indomethacin, a non-selective cyclo-oxygenase (COX) inhibitor, resulted in a downward shift in baseline tone, suggesting inhibition of the contractile effects of SBE (Fig. 1D). This observation may indicate that in rabbit isolated aortic ring preparations, the SBE-induced contractile responses may have occurred either via the COX-1 or COX-2 pathways, which are responsible for releasing vasoconstrictors such as prostaglandin F_2_, prostaglandin E and endothelin-1.^16^ Inhibition of the COX pathway with indomethacin would therefore promote relaxation, as with NO.

The magnitude of the contractile effects of SBE was slightly less in the endothelium-denuded aortic rings than in the intact rings (Fig. 1B). This could be due to the absence of powerful vasoconstrictors such as endothelins, prostanoids and prostaglandins, which are normally expressed in intact, functional endothelium.

The ability of verapamil, an L-type calcium channel blocker, to partially inhibit the SBE-induced contraction of the rabbit aortic ring preparations may indicate involvement of calcium ions in the contractile responses (Fig. 1E). This suggests that SBE may have caused membrane depolarisation, thus facilitating mobilisation of Ca^2+^ from the extracellular fluid into the muscle cells via the L-type voltage-gated calcium channels.

Alternatively, SBE could have promoted the accumulation of intracellular Ca^2+^ by inhibition of the Na^+^/Ca^2+^ exchangers or the Na^+^/K^+^ channels, which remove calcium from the cells. Ca^2+^-free treatment of the rabbit aortic ring preparations, however, had no visible effect when SBE was added to the bath fluid. This suggests the absence of SBE-induced intracellular Ca^2+^ release in the rabbit preparations, perhaps due to diminished influx of extracellular calcium.

The findings of the present study indicated that SBE also caused contractile effects on rat isolated portal veins. Graded concentrations of SBE raised the basal tone, and subsequently caused marked concentration-dependent increases in the amplitudes of the myogenic contractions of the venous tissue, with an EC_50_ value of 36 ± 6 mg/ml (Fig. 2B, C). SBE-induced contractions were not modified by pre-incubation of the tissues with prazosin, suggesting that the contractile responses of the venous tissue were unlikely to have been mediated via α_1_-adrenoceptor stimulation (Fig. 2E).

Pre-treating the animals with reserpine, a drug that depletes catecholamines from tissue stores, had no effect on the SBE-induced contractions of the venous tissue (Fig. 2F). This may imply that the SBE-induced contractile responses of the venous tissue were unlikely to have been mediated via catecholamine release from tissue stores.

Pre-incubation of the portal vein preparations in calcium-free Krebs-Henseleit solution resulted in contractile responses after SBE addition. The contractions were completely reversed by washing out the SBE solutions (Fig. 2D). However, SBE was unable to reverse the verapamil-induced suppression of the rat portal vein contractility. This could imply that SBE acted as an agonist for intracellular Ca^2+^ release, but this required an influx of extracellular calcium, which was blocked by verapamil. Removal of the agonist (SBE) therefore resulted in the termination of Ca^2+^ release from the intracellular stores.

SBE has been reported to contain a number of chemical compounds, including tannins, polyphenols, coumarins, triterpenoids and phytosterols.^7^,^10^,^17^ The flavonoids present in *S birrea* leaves include quercetin and kaempferol, as well as their esters, gallic acid, myricetin and catechin gallates, such as (-)-epigallocatechin 3-*O*-galloyl ester and (-)-epicatechin 3-*O*-galloyl ester.^18^ Although the exact chemical constituents of SBE responsible for the observed contractile effects of the plant extract on mammalian isolated vascular tissue remain speculative at present, the synergistic effects of (-)-epigallocatechin 3-*O*-galloyl, (-)-epicatechin 3-*O*-galloyl, gallic acid and myricetin have previously been reported.^7^,^17^,^19^

The results obtained in this study suggest that SBE-induced contraction of smooth muscles may have been mediated via activation of the cyclo-oxygenase pathways. In a study by Gil-Longo and Gonzalez-Vazquez,^19^ pre-incubating smooth muscle with indomethacin practically abolished the contractile effects of gallic acid. This was attributed to the generation of free radicals, which inactivate endothelial resting vasodilator NO.

The flavonoids commonly found in SBE, myricetin and quercetin, have previously been reported to elicit smooth muscle contraction. Myricetin has been observed to elicit smooth muscle contraction via activation of the phospholipase-A_2_ pathway, resulting in the release of thromboxane A_2_ by increasing Ca^2+^ concentrations.^20^

Quercetin has been identified as a novel, specific activator of L-type calcium channels^21^ and it increases the influx of Ca^2+^,^21^ which contradicts its well-known vasodilatory effects.^22^ The myorelaxant effect of quercetin in tissue preparations has been suggested to originate from its reaction with a second target beyond the Ca^2+^ channel.^21^ In the present study, however, it may be argued that the SBE-induced contractile effects were likely to have been a result of the sudden influx of calcium into the cells, due to the quercetin activation of L-type calcium channels. It can therefore be speculated that the pharmacological effects observed with SBE in our experiments may in part have been due to a synergistic effect of the flavonoids present in SBE.

## Conclusion

The findings of this study show that SBE exerted contractile effects on isolated vascular smooth muscles *in vitro*. The SBE-induced vasoconstriction may have been mediated, at least in part, by the actions of endothelium-derived vasoconstrictors such as prostaglandins and endothelin-1, as well as by the influx of extracellular Ca^2+^ and release of intracellular Ca^2+^. These observations suggest that SBE may increase blood pressure; hence it may exacerbate hypertension in vulnerable patients. Further *in vivo* investigations are suggested.
